# From health to periodontitis: dynamic changes in the subgingival microbiome and their association with systemic inflammation levels

**DOI:** 10.3389/fcimb.2026.1864541

**Published:** 2026-06-19

**Authors:** Yan Liu, Chunshen Li, Yunshan Zhao, Liang-qiu-yue Zhong, Yi Li, Zhongyi Xiao, Qifeng Liu, Xi Chen

**Affiliations:** Department of Stomatology, The First Affiliated Hospital of Xi’an JiaoTong University, Xi’an, Shaanxi, China

**Keywords:** 16S rRNA gene sequencing, microbial succession, periodontitis, subgingival microbiome, systemic inflammation

## Abstract

**Introduction:**

This study aims to characterize the dynamic progression of the subgingival microbiome across different stages of periodontitis and to explore its association with levels of systemic chronic inflammation.

**Methods:**

A total of 148 subjects were enrolled based on predefined inclusion and exclusion criteria. Participants were classified into five groups according to diagnostic criteria: Stage I (n = 25), Stage II (n = 30), Stage III (n = 31), Stage IV (n = 30), and a healthy control group (n = 32). Subgingival samples were collected from all participants and subjected to the 16S rRNA gene sequencing. Peripheral venous blood was obtained to determine blood cell counts and to calculate systemic inflammatory markers. The Spearman’s correlation analysis was performed to evaluate associations between subgingival microbial communities and systemic inflammatory markers.

**Results:**

Patients with Stage IV periodontitis exhibited significantly higher levels of WBC, NEUT, NLR, and SII compared to those with Stage I disease. Notably, NLR and SII were markedly elevated (*P* < 0.01), while WBC and NEUT also showed statistically significant increases (*P* < 0.05). Further analysis revealed a positive correlation between the abundance of multiple periodontitis-associated bacterial genera and systemic inflammatory markers.

**Discussion:**

This study demonstrates that the progression of periodontitis is associated with distinct changes in the subgingival microbial community, and that this microbial dysbiosis is positively correlated with elevated levels of systemic chronic inflammation.

## 1 Introduction

The oral microbiome represents the second-largest microbial community in the human body, following the gut. It exhibits a complex and highly diverse structure, primarily comprising bacterial, fungal, and viral components. (Mi et al., 2024) Among these, bacteria have been the most extensively characterized, while fungi and viruses-owing to relatively limited research-are often regarded as minor constituents of the oral ecosystem. (Lin et al., 2024) Under healthy conditions, the oral microbiome maintains a dynamic equilibrium with the host, collectively ensuring oral homeostasis. However, disruption of this balance, termed oral dysbiosis, leads to alterations in microbial composition and function, which may subsequently trigger local diseases such as dental caries and periodontitis. (Belstrøm et al., 2017) Periodontitis is a prevalent inflammatory oral disease characterized by progressive destruction of periodontal tissues, including the gingiva, periodontal ligament, and alveolar bone. (Abusleme et al., 2021) In severe cases, it can result in tooth loss, substantially impairing patients’ quality of life and imposing a considerable economic burden. (Conceição et al., 2025) The primary pathogens involved are predominantly anaerobic Gram-negative bacteria, which, through the expression of various virulence factors, induce dysregulation of local and systemic inflammatory mediators. (Stojilković et al., 2023) This not only drives the progression of local periodontal lesions but may also influence systemic health via multiple pathways, contributing to the initiation and development of various systemic diseases. (Zhu et al., 2025) Despite continuous advancements in medical technology, it is estimated that approximately 10%-15% of the global population continues to be affected by severe periodontitis. (Kassebaum et al., 2014) This persistent prevalence underscores current deficiencies in early detection and intervention, highlighting the urgent clinical need for the development of novel biomarkers and therapeutic targets.

It is now widely accepted that the pathogenesis of periodontitis arises from the synergistic effects of the subgingival microbial community and its ecological dysbiosis. (Iniesta et al., 2023) The onset and progression of this disease are influenced not only by specific pathogens but also by the overall ecological imbalance of the subgingival microbiota. (Ortiz et al., 2022) As periodontitis progresses from mild to severe stages, the enrichment of pathogenic microorganisms follows a characteristic succession pattern, involving shifts in both the composition and abundance of multiple microbial species. In susceptible individuals, these bacteria act synergistically to drive local inflammation and tissue destruction. (Wang et al., 2025) In recent years, advances in high-throughput sequencing technologies have enabled detailed characterization of the subgingival microbial community. Elucidating structural changes in the microbiota across different disease stages is essential for understanding the pathogenesis of periodontitis. (Xu et al., 2025) However, further investigation is needed to clarify the dynamic succession patterns of the subgingival microbiome during disease progression and their relationship with clinical manifestations. Notably, periodontitis is not confined to the oral cavity; it may also increase the risk of various systemic diseases, such as myocardial infarction, (Chen et al., 2025) preterm birth, (Couceiro et al., 2025) chronic obstructive pulmonary disease (COPD), (Xiong et al., 2023) stroke, (Meng and Chen, 2025) and rheumatoid arthritis by exacerbating systemic inflammatory responses. (Lopez‐Oliva et al., 2025) The potential mechanisms underlying this association primarily involve two pathways: infectious (direct effects) and inflammatory (indirect effects). The inflammatory pathway posits that periodontal pathogens, along with locally produced inflammatory mediators, are continuously released into the bloodstream from damaged gingival tissue, thereby activating the immune system and inducing systemic low-grade inflammation. (Plachokova et al., 2021; Ren et al., 2024) Nevertheless, systematic research on the association between subgingival microbial dysbiosis in periodontitis and systemic inflammation remains limited. A comprehensive analysis of the mechanisms linking the subgingival microbiome to systemic diseases will not only advance our understanding of the systemic effects of periodontitis but also provide new insights for the development of early warning and intervention strategies based on microbial biomarkers.

Based on the aforementioned research background, this study enrolled a total of 148 participants, including periodontally healthy individuals and patients with periodontitis at various stages. By employing 16S rRNA sequencing to analyze subgingival plaque samples, in conjunction with blood-based measurements of systemic inflammatory markers, this study aims to systematically characterize the compositional features and succession patterns of the subgingival microbiome across different stages of periodontitis. Furthermore, it seeks to explore the potential associations among subgingival microbial dysbiosis, periodontitis progression, and systemic inflammation level. The findings of this study are expected to provide novel insights into the interactions between the subgingival microbiota and the host immune system during the progression of periodontitis. They may further elucidate the correlations between specific periodontitis-associated microbial communities and systemic inflammatory responses, thereby laying a scientific foundation for the development of risk assessment and early intervention strategies for systemic diseases based on the subgingival microbiome.

## 2 Materials and methods

### 2.1 Study population and grouping

**Figure 1:**
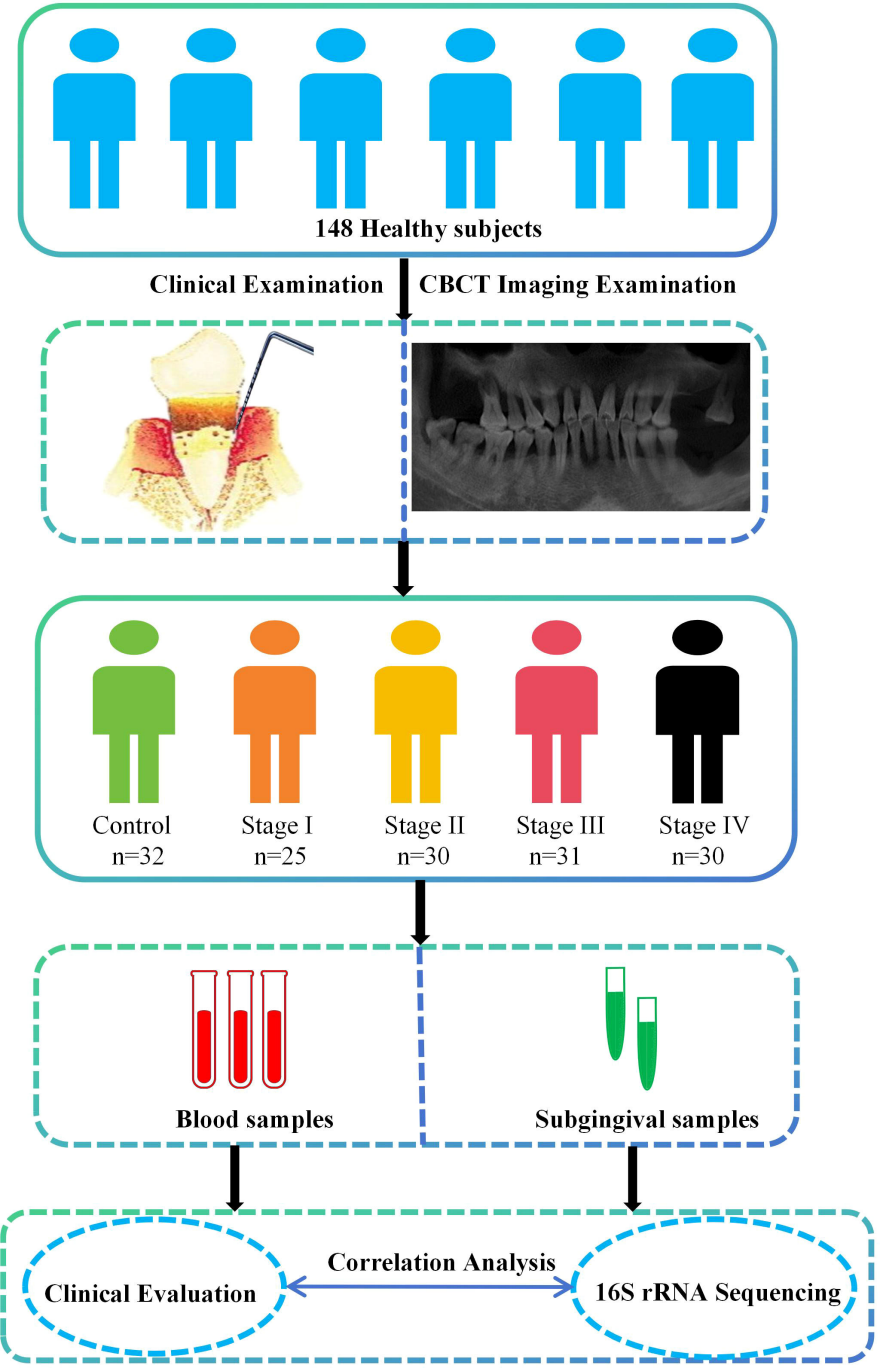
The flowchart of this study showing the enrollment and sample collection, as well as the subsequent analysis of the correlation between the microbiome and systemic inflammatory markers.

This study was approved by the Research Ethics Committee of the First Affiliated Hospital of Xi’an Jiaotong University (XJTU1AF2024LSYY-480). All subjects signed informed consent forms prior to study participation. A total of 148 systemically healthy participants were recruited from the Department of Stomatology, the First Affiliated Hospital of Xi’an Jiaotong University. The inclusion criteria were as follows: (1) presence of more than 20 teeth; (2) no history of periodontal or orthodontic treatment within the preceding 6 months; (3) absence of oral diseases other than periodontitis (e.g., severe dental caries, periapical pathology, or oral mucosal diseases); (4) no medication use in the past 6 months; (5) absence of systemic diseases (e.g., hematological disorders, immune system diseases, infectious diseases, diabetes mellitus, atherosclerotic cardiovascular disease, or other systemic conditions); (6) not pregnant or breastfeeding; (7) no history of smoking; (8) body mass index (BMI) ≤ 28 kg/m²; (9) age between 18 and 55 years. Based on clinical examination and radiographic findings, participants were divided into a control group (n = 32) and a periodontitis group (n = 116). Periodontitis was further classified into Stages I, II, III, and IV according to according to the new diagnostic criteria proposed at the 2017 AAP and EFP World Symposium. The control group was characterized by the following: (1) bleeding on probing (BOP) < 10%; (2) periodontal probe depth (PPD) ≤ 3 mm; (3) no clinical attachment loss (CAL); (4) no radiographic evidence of alveolar bone resorption; (5) no history of periodontitis. The flowchart illustrating the study design and sample collection is presented in **Figure 1**.

### 2.2 Clinical examination

This study conducted comprehensive periodontal examinations on all participants, including periodontal probe depth (PPD), clinical attachment level (CAL), and bleeding on probing (BOP). Cone-beam computed tomography (CBCT) scans were performed on enrolled subjects to assess radiographic bone loss (RBL). Additionally, demographic characteristics including age, gender, height, and weight were recorded, and the body mass index (BMI) was calculated in terms of height and weight. All clinical examinations were performed by two calibrated periodontists who were unaware of the participants’ clinical grouping.

### 2.3 Sample collection and preparation

Subgingival samples were collected prior to clinical examination from the following sites: the first molars on the right maxilla and left mandible, the first premolars on the left maxilla and right mandible, the central incisors on the left maxilla and right mandible. Before sample collection, the selected teeth were isolated with sterile cotton rolls and gently air-dried. After removal of supragingival plaque, subgingival plaque was obtained using a Gracey curette and immediately placed into sterile Eppendorf tubes containing 1.5 mL of 1× phosphate-buffered saline (pH =7.2). All samples were stored at -80 °C until further processing. Samples with visible blood contamination were excluded from the study.

Fasting venous blood samples (5 mL) were collected from all participants between 8:00 and 10:00 a.m. using sodium citrate anticoagulant tubes. The samples were immediately transported to the Department of Laboratory Medicine at the First Affiliated Hospital of Xi’an Jiaotong University for complete blood cell analysis using an automated hematology analyzer (Sysmex HST-302, Japan). The following parameters were measured: lymphocyte count (LYMPH), monocyte count (MONO), neutrophil count (NEUT), and white blood cell count (WBC). Based on these measurements, the following inflammatory indices were calculated: neutrophil-to-lymphocyte ratio (NLR), platelet-to-lymphocyte ratio (PLR), lymphocyte-to-monocyte ratio (LMR), and systemic immune-inflammation index (SII). The SII is a composite index derived from platelet, neutrophil, and lymphocyte counts. All blood samples were collected prior to any periodontal treatment.

### 2.4 Microbial DNA extraction, PCR amplification, and sequencing

Total genomic DNA was extracted from subgingival plaque samples using the OMEGA DNA Kit (USA) according to the manufacturer’s instructions. For microbial community analysis, the V3–V4 hypervariable regions of the 16S rRNA gene were amplified by polymerase chain reaction (PCR) using the forward primer CCTACGGGNGGCWGCAG and the reverse primer GACTACHVGGGTWTCTAATCC. PCR amplification was performed under the following conditions: initial denaturation at 98 °C for 30 s, followed by 32 cycles of denaturation at 98 °C for 10 s, annealing at 54 °C for 30 s, and extension at 72 °C for 45 s, with a final extension at 72 °C for 10 min. PCR products were purified using AMPure XP beads (Beckman Coulter Genomics, Danvers, MA, USA) and quantified using a Qubit fluorometer (Invitrogen, USA). The purified amplicons were then evaluated using the Illumina library quantification kit (Kapa Biosciences, Woburn, MA, USA), with only libraries meeting the concentration threshold of ≥ 2 nM proceeding to sequencing. The Sequencing was performed on the DNBSEQ-G99RS platform using the G99 App-DFCL PE300 high-throughput sequencing kit. Qualified libraries (ensuring no duplicate index sequences) were serially diluted, pooled in appropriate proportions according to the required sequencing volume, and denatured with sodium hydroxide to generate single-stranded DNA. The single-stranded DNA fragments were then hybridized to the Illumina-specific primers immobilized on the sequencing chip and subjected to bridge amplification. For sequencing, Read1 sequencing primers were added along with engineered DNA polymerase and four-color fluorescently labeled dNTPs. Single-base extension was performed in each cycle, and the corresponding fluorescent signals were captured to determine the nucleotide sequence. Following Read1 completion, the synthesized strands were washed away, and index primers were added for index sequencing. After index sequencing, reverse bridge amplification was performed, followed by addition of Read2 sequencing primers for paired-end sequencing. Raw data were generated and filtered by the sequencer according to the corresponding algorithms.

Following sequencing, the paired-end reads were demultiplexed and subjected to quality control and filtering based on sequencing quality metrics. The high-quality reads were then assembled according to their overlapping regions to generate optimized data. This processed dataset was subsequently used for clustering analysis and taxonomic classification. Based on the clustering results, further analyses were performed, including species composition profiling, alpha diversity analysis, beta diversity analysis, and identification of differentially abundant species.

### 2.5 Statistical analysis

The experimental data were analyzed using IBM SPSS Statistics 27 and GraphPad Prism 9.0. Continuous variables are expressed as mean ± standard deviation. For continuous variables, normality was first assessed using the Shapiro–Wilk test when the sample size was ≤ 50. *P* < 0.05 was considered indicative of non-normal distribution. Comparisons of continuous variables among multiple groups were performed using the Kruskal–Wallis. Given the non-normal distribution of the continuous variables, Spearman’s correlation analysis was employed to examine the relationships among various parameters in different groups at baseline.

Alpha diversity indices were employed to estimate the richness, evenness, and diversity of microbial communities. Specifically, the Chao1 index was used to assess microbial community richness, reflecting the number of species present. The Shannon index was applied to evaluate community diversity, integrating both species richness and evenness. Pielou’s evenness index characterized species evenness, with higher values indicating more even distribution. Faith’s phylogenetic diversity (Faith’s PD) index was utilized to explore the relationship between species abundance and evolutionary distance, where higher values denote greater community diversity. The Beta diversity was analyzed using principal coordinate analysis (PCoA) based on Bray-Curtis distance and weighted UniFrac distance. By reducing data dimensionality, this approach elucidated similarities or differences in microbial community composition among samples. In conjunction with non-parametric permutational multivariate analysis of variance (PERMANOVA), it assessed whether differences in microbial community composition between sample groups were statistically significant. Linear discriminant analysis effect size (LEfSe) was performed to identify differentially abundant taxa. This method first employs non-parametric Kruskal–Wallis to detect features with significant differences, followed by linear discriminant analysis (LDA) to estimate effect sizes. Bacterial taxa exhibiting significant differences in abundance at the genus level among groups were identified using a threshold of LDA score ≥ 4 and *P* < 0.05.

## 3 Results

### 3.1 Demographic and clinical characteristics of the population

A total of 148 participants were included in this study, comprising 25 patients with Stage I periodontitis, 30 with Stage II periodontitis, 31 with Stage III periodontitis, 30 with Stage IV periodontitis, and 32 healthy controls. No significant differences were observed among the five groups regarding age, gender, and BMI (*P* > 0.05), indicating successful group matching. Regarding periodontal clinical parameters, patients with Stage III and Stage IV periodontitis exhibited significantly higher PPD and BOP (%) compared to those with Stage I and Stage II periodontitis (*P* < 0.001). For CAL, patients with Stage IV periodontitis showed significantly greater values than those in the Stage I and Stage II groups (*P* < 0.001). Additionally, patients with Stage III periodontitis demonstrated significantly higher CAL compared to both Stage I (*P* < 0.001) and Stage II (*P* < 0.05) groups. Detailed demographic and clinical characteristics are presented in **Table 1**.

**Table 1:** Demographic and clinical characteristics of participants.

Demographics	Control (n=32)	Stage			
		I (n=25) mean ± SD	II (n=30) mean ± SD	III (n=31) mean ± SD	IV (n=30) mean ± SD
Age, mean ± SD (years)	33.94 ± 7.36	33.68 ± 6.79	36.47 ± 8.76	39.03 ± 8.39	39.13 ± 8.40
Male/Female	17/15	11/14	16/14	14/17	13/17
BMI, mean ± SD (kg/m^2^)	23.16 ± 2.09	23.20 ± 1.97	23.53 ± 1.90	23.64 ± 1.54	23.35 ± 4.76
Periodontal indices					
PPD, mean ± SD (mm)	1.94 ± 0.62	2.44 ± 0.51^a^	3.40 ± 0.56^cf^	4.19 ± 0.60^cf#^	4.53 ± 0.57^cf#^
CAL, mean ± SD (mm)	–	1.88 ± 0.33	3.23 ± 0.43	4.32 ± 0.48^f&^	4.83 ± 0.75^f#^
RBL (%) mean ± SD	–	9.45 ± 2.05	22.93 ± 3.28	38.07 ± 5.82^f^	52.87 ± 2.53^f#^
BOP (%) mean ± SD	8.67 ± 4.60	38.9 ± 9.79	64.50 ± 11.11^c^	88.91 ± 4.88^cf#^	90.71 ± 3.59^cf#^

“a” indicates *P <* 0.05 compared with the healthy control group; “b” indicates *P* < 0.01 compared with the healthy control group; “c” indicates *P* < 0.001 compared with the healthy control group; “d” indicates *P* < 0.05 compared with the stage I; “e” indicates *P* < 0.01 compared with the stage I; “f” indicates *P* < 0.001 compared with the stage I; “&” indicates *P* < 0.05 compared with the stage II; “#” indicates *P* < 0.001 compared with the stage II. The number “n” in parentheses represents the number of cases.

### 3.2 Changes in systemic inflammatory markers in patients with periodontitis at different stages

Compared with the Stage I periodontitis group, patients with Stage IV periodontitis exhibited elevated levels of WBC, NEUT, NLR, and SII. Among these, NLR and SII showed significant increases (*P* < 0.01), while WBC and NEUT also demonstrated statistically significant differences (*P* < 0.05). Furthermore, NEUT levels were significantly higher in patients with Stage IV periodontitis than in those with Stage II periodontitis (*P* < 0.05). No statistically significant differences were observed for the remaining indicators among the groups (*P* > 0.05). The results are presented in **Table 2**.

**Table 2:** Changes in systemic inflammatory markers in patients with periodontitis.

Systemic inflammatory markers	Control (n=32)	Stage			
		I (n=25) mean ± SD	II (n=30) mean ± SD	III (n=31) mean ± SD	IV (n=30) mean ± SD
WBC (×10^9^/L) mean ± SD	5.53 ± 0.53	5.69 ± 0.79	5.85 ± 0.90	6.15 ± 0.91^a^	6.22 ± 0.67^bd^
NEUT (×10^9^/L) mean ± SD	3.15 ± 0.62	3.37 ± 0.62	3.55 ± 0.94	3.87 ± 1.03^a^	4.12 ± 0.75^cd&^
NLR mean ± SD	1.59 ± 0.48	1.65 ± 0.44	2.01 ± 0.80	2.19 ± 0.89^a^	2.29 ± 0.59^ce^
PLR mean ± SD	121.29 ± 38.39	123.08 ± 30.80	138.09 ± 46.11	141.11 ± 57.00	151.44 ± 42.29^a^
LMR mean ± SD	6.27 ± 2.17	6.48 ± 1.82	5.20 ± 1.77	5.36 ± 2.05	5.42 ± 1.47
SII mean ± SD	384.16 ± 148.29	420.98 ± 151.55	480.86 ± 178.77	541.82 ± 245.01^a^	625.88 ± 206.76^ce^

“a” indicates *P <* 0.05 compared with the healthy control group; “b” indicates *P* < 0.01 compared with the healthy control group; “c” indicates *P* < 0.001 compared with the healthy control group; “d” indicates *P* < 0.05 compared with the stage I; “e” indicates *P* < 0.01 compared with the stage I; “f” indicates *P* < 0.001 compared with the stage I; “&” indicates *P* < 0.05 compared with the stage II; “#” indicates *P* < 0.001 compared with the stage II. The number “n” in parentheses represents the number of cases.

### 3.3 Changes in the alpha and beta diversity of the subgingival microbial community in patients with periodontitis at different stages

**Figure 2:**
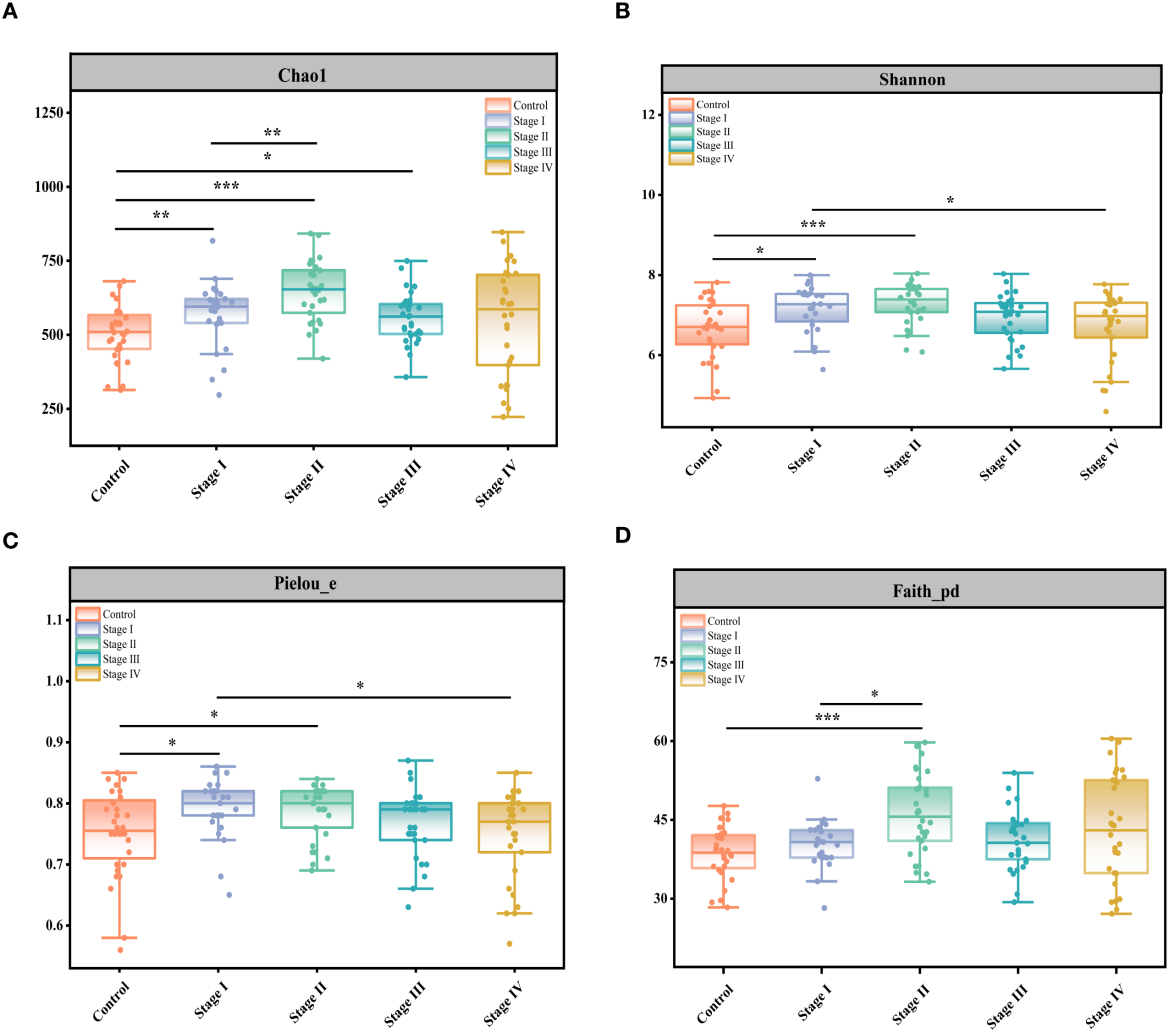
The results of alpha diversity among the five groups. **(A)** The Chao1 index; **(B)** The Shannon index; **(C)** The Pielou’s index; **(D)** The Faith’s pd index. *P<0.05, **P<0.01, ***P<0.001.

Alpha diversity indices, including Chao1, Shannon, Pielou’s evenness (Pielou’s e), and Faith’s phylogenetic diversity (Faith’s pd), were employed to assess microbial community richness, diversity, and evenness. As periodontal disease severity increased, alpha diversity exhibited distinct trends (**Figure 2**). Notably, the control group demonstrated the lowest alpha diversity indices among all groups. Specifically, the Chao1 index in the control group was significantly lower than that in the Stage I (*P* < 0.01), Stage II (*P* < 0.001), and Stage III (*P* < 0.05) periodontitis groups, while no significant difference was observed compared with the Stage IV group (*P* > 0.05) in **Figure 2A**. The Shannon index, reflecting community diversity, was lower in the control group than in the Stage I (*P* < 0.05) and Stage II (*P* < 0.001) periodontitis groups, although no significant differences were found compared with the Stage III or Stage IV groups (both *P* > 0.05), as shown in **Figure 2B**. Regarding evenness, the Pielou’s e index in the control group was significantly lower than that in the Stage II periodontitis group (*P* < 0.05), but showed no significant differences compared with the Stage I, Stage III, or Stage IV groups (*P* > 0.05), as shown in **Figure 2C**. For phylogenetic diversity, the Faith’s pd index in the control group was significantly lower than that in the Stage II periodontitis group (*P* < 0.001), while no significant differences were observed compared with the Stage I, Stage III, or Stage IV groups (*P* > 0.05), as shown in **Figure 2D**. These trends were consistent with the rarefaction curve analyses.

Alpha diversity reflects both species richness (abundance) and evenness (distribution). The observation of higher species richness in periodontitis groups compared with healthy controls aligns with the current understanding that subgingival dysbiosis results from microbial succession rather than simple replacement of initial colonizers. Notably, the Stage II periodontitis group exhibited the highest levels of both specie richness and diversity among all groups. Although microbial richness remained relatively elevated in Stage IV periodontitis patients-indicating that substantial species loss had not yet occurred at this stage-certain microbial taxa appeared to become dominant within the periodontitis-associated community (i.e., their relative proportions increased). This shift led to reduced community evenness and, consequently, lower overall diversity compared with patients at earlier stages of periodontitis.

**Figure 3:**
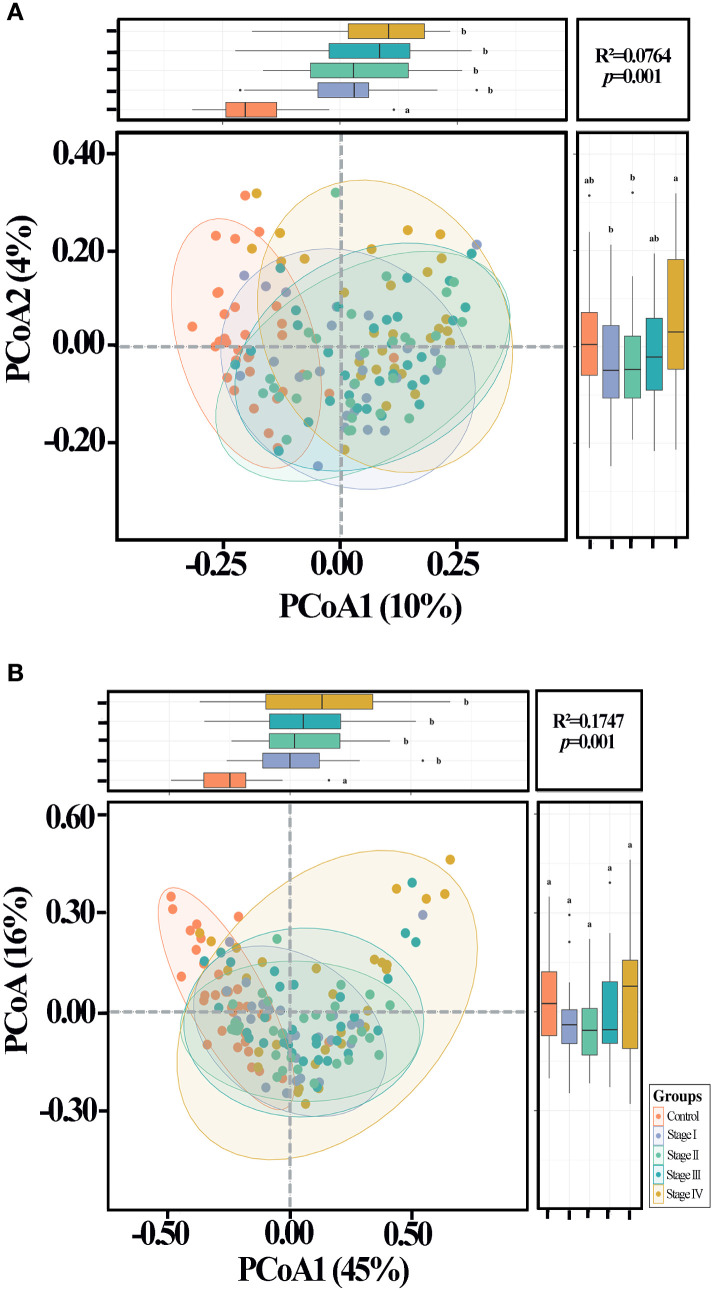
The results of beta diversity among the five groups. **(A)** PCoA analysis based on Bray-Curtis distance; **(B)** PCoA analysis based on weighted UniFrac distance. The results of the PERMANOVA analysis are indicated in the upper right corner of each Beta diversity PCoA.

Beta diversity analysis was performed to compare microbial community composition among different samples. PCoA was employed to explore differences in subgingival microbial community structure between patients with periodontitis at different stages and healthy controls. In the PCoA plots, different colors represent distinct groups, with each point within the same color indicating an individual sample from that group. Ellipses denote 95% confidence intervals for each group. The distance between samples reflects the degree of dissimilarity in microbial composition, with shorter distances indicating greater similarity and longer distances indicating greater dissimilarity. Permutational multivariate analysis of variance (PERMANOVA) results are displayed in the upper right corner of each plot, where the R² value represents the proportion of variation explained by group differences, and a *P* < 0.05 indicates statistically significant differences between groups. As shown in **Figure 3**, PCoA based on both Bray-Curtis distance (which considers species abundance) and weighted UniFrac distance (which incorporates phylogenetic information) revealed a trend toward separation among the five groups. In **Figure 3A**, analysis using Bray-Curtis distance with PERMANOVA demonstrated significant differences in microbial community structure among the five groups (R² = 0.0764, *P* = 0.001). Similarly, **Figure 3B** shows that evaluation using Weighted UniFrac distance also revealed statistically significant differences (R² = 0.1747, *P* = 0.001). Both beta diversity metrics consistently indicated significant differences in subgingival microbial community structure among patients with periodontitis at different stages and healthy individuals. Although some overlap was observed among the five groups, they were generally clustered separately, with intergroup differences exceeding intragroup differences, and these differences were statistically significant (*P* = 0.001). Notably, the Stage IV periodontitis group exhibited greater variability in microbial community structure, suggesting that the onset and progression of periodontitis are associated with distinct shifts in the subgingival microbiome.

### 3.4 Composition and structure of the subgingival microbial community in patients with periodontitis at different stages

**Figure 4:**
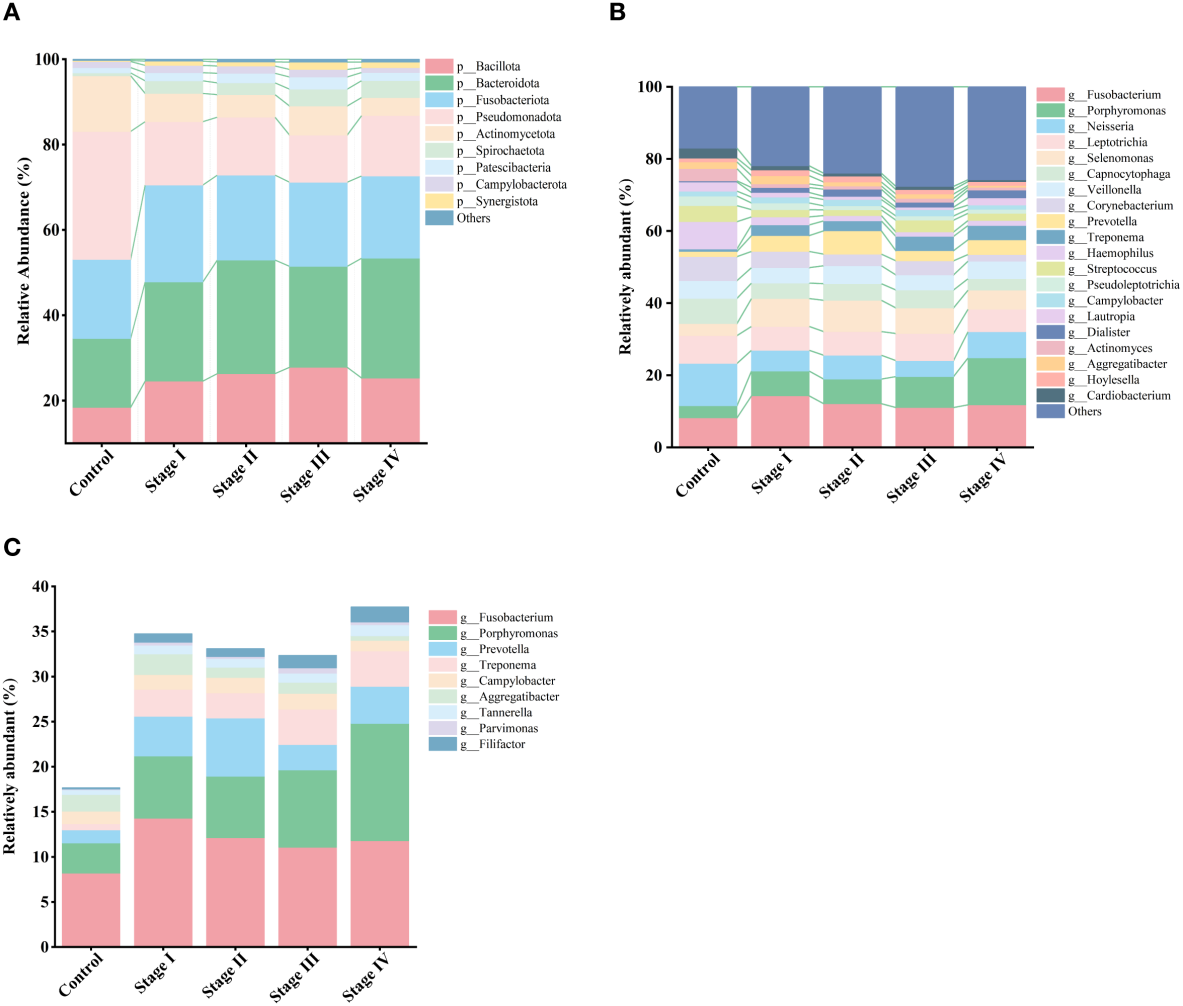
The relative abundances of microbial genera among the five groups. **(A)** Distribution of subgingival bacterial communities at the phylum level across groups; **(B)** Distribution of subgingival bacterial communities at the genus level across groups; **(C)** Relative abundances of genera associated with major periodontal pathogens.

At the phylum level, the predominant bacterial taxa in terms of relative abundance were largely consistent across the five groups, with the most abundant phyla being *Bacillota*, *Bacteroidota*, *Fusobacteria*, *Pseudomonadota*, and *Actinobacteriota*. However, notable shifts in relative abundance were observed as periodontitis progressed. Specifically, the relative abundance of *Bacteroidota* increased progressively from 16% in the control group to 28% in the Stage IV periodontitis group. In contrast, the abundance of *Pseudomonadota* decreased markedly from 30% to 14% over the same progression in **Figure 4A**. At the genus level, the relative abundances of *Porphyromonas* (increasing from 3% to 13%) and *Treponema* (increasing from 1% to 4%) exhibited progressive increases from the control group through to the Stage IV periodontitis group. Conversely, the abundances of *Haemophilus* (decreasing from 7% to 1%), *Cardiobacterium* (decreasing from 3% to 1%), and *Actinomyces* (decreasing from 3% to 1%) showed gradual declines with deteriorating periodontal health in **Figure 4B**.The total abundance of the red complex, which comprises the major periodontal pathogens *Porphyromonas, Tannerella*, and *Treponema*, progressively increased across the control group and the periodontitis groups (Stages I, II, III, and IV), reaching relative abundances of 5%, 11%, 11%, 13%, and 18% respectively. In contrast, the total abundance of the orange complex, consisting of *Fusobacterium*, *Prevotella, Parvimonas*, and *Campylobacter, i*ncreased substantially from 11% in the healthy control group to 21% in the Stage I periodontitis group, remained elevated at 21% in the Stage II group, but subsequently decreased to 16% and 17% in the advanced Stage III and Stage IV groups respectively in **Figure 4C**.

### 3.5 Differences of the subgingival microbial community in patients with periodontitis at different stages

**Figure 5:**
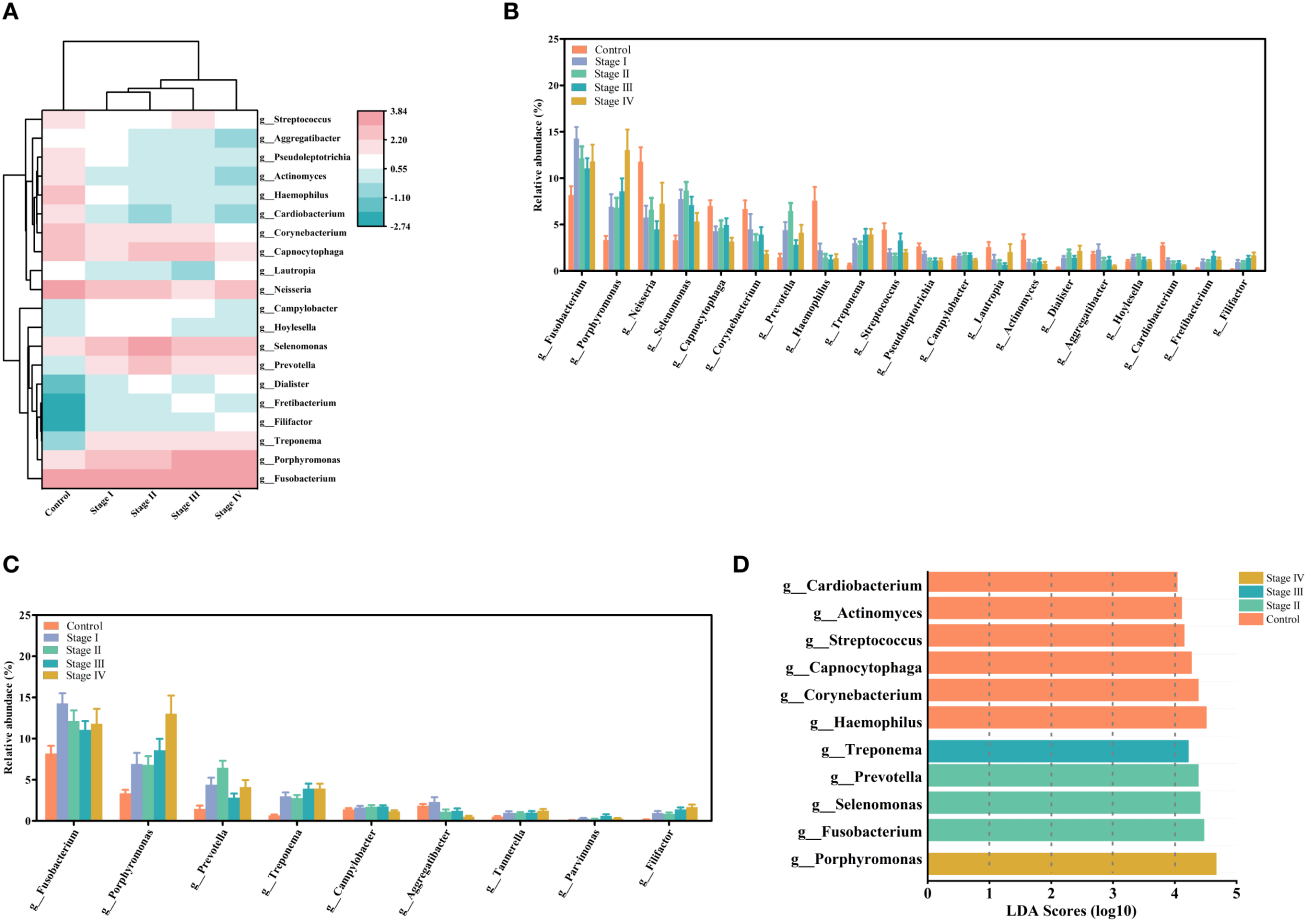
The differential distribution of subgingival bacterial genera among the five groups. **(A)** Clustering heatmap showing the top 20 bacterial genera with significant differences in relative abundance across the five groups; **(B)** Box plots illustrating the relative abundances of the top 20 differentially abundant bacterial genera among groups; **(C)** Intergroup comparison of periodontal pathogen-associated bacterial genera; **(D)** Subgingival microbial communities with significant differences across groups; the higher the LDA score, the greater the influence of species abundance on the effect of differences. The Kruskal-Wallis was employed to compare bacterial genera with significant differences across groups.

**Figure 5A** presents the top 20 genera with significant differences in relative abundance among the study groups. The heatmap revealed two distinct clusters: the left cluster predominantly comprised samples from the control group, while the right cluster consisted of samples from patients with Stage I, II, III, and IV periodontitis. Marked differences in dominant genera were observed between the two clusters. The left cluster exhibited higher abundances of *Haemophilus, Streptococcus, Neisseria, Actinomyces, Capnocytophaga*, and *Corynebacterium*. In contrast, the right cluster showed higher abundances of *Treponema, Prevotella, Porphyromonas*, and *Fusobacterium.* Between group comparisons at the genus level revealed significant differences among the five groups. Among the 20 most relatively abundant genera with significant intergroup differences, the control group demonstrated higher abundances of *Haemophilus, Corynebacterium, Actinomyces, Leptotrichia, Cardiobacterium, Neisseria*, and *Streptococcus* compared to the periodontitis groups. Conversely, the periodontitis groups exhibited higher abundances of *Porphyromonas, Dialister, Prevotella, Fusobacterium, Selenomonas, Tannerella, Fretibacterium*, and *Treponema* relative to the control group in **Figure 5B**. We further examined nine genera closely associated with periodontal pathogenesis-key drivers of subgingival dysbiosis and inflammation in periodontitis. The relative abundances of all nine genera differed significantly among the five groups (*P* < 0.05). In the periodontitis groups, *Porphyromonas, Tannerella*, and *Treponema* were consistently elevated. Notably, *Prevotella* and *Fusobacterium* reached their highest abundances in the Stage II periodontitis group, whereas *Porphyromonas, Tannerella*, and *Filifactor* peaked in the Stage IV periodontitis group in **Figure 5C**. Linear discriminant analysis effect size (LEfSe) was performed to identify differentially abundant taxa among groups (LDA ≥ 4), revealing potential microbial biomarkers for each group. As shown in **Figure 5D**, the control group was significantly enriched in *Capnocytophaga, Corynebacterium, Cardiobacterium, Streptococcus*, and *Actinomyces*. The Stage II periodontitis group showed significant enrichment of *Fusobacterium*, *Prevotella*, and *Selenomonas*. The Stage III periodontitis group was characterized by significant enrichment of Treponema, while the Stage IV periodontitis group exhibited significant enrichment of *Porphyromonas*.

**Figure 6:**
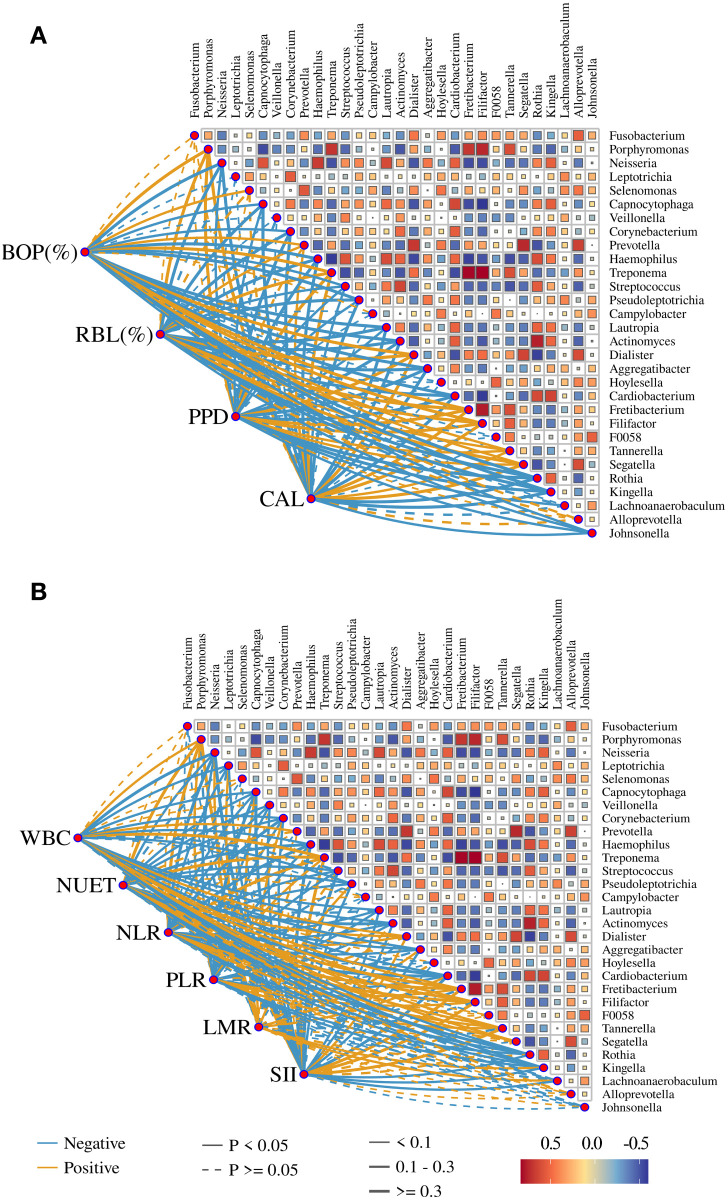
The correlation analysis of subgingival microbial genera with periodontal clinical parameters and systemic inflammatory markers. **(A)** Heatmap showing correlations between subgingival bacterial genera and periodontal clinical parameters across groups; **(B)** Heatmap illustrating correlations between subgingival bacterial genera and systemic inflammatory markers across study groups. Red indicates positive correlations, while blue indicates negative correlations.

The correlation analysis was performed to examine the associations between periodontal clinical parameters and the relative abundance of the top 30 bacterial genera. The results revealed that 20 bacterial genera were significantly correlated with PPD (*P* < 0.05), including seven with positive correlations and 13 with negative correlations. Bacterial genera exhibiting a correlation coefficient |r| ≥ 0.3, listed in descending order of correlation strength, were as follows: *Rothia* (r = -0.48), *Cardiobacterium* (r = -0.46), *Haemophilus* (r = -0.43), *Actinomyces* (r = -0.42), *Filifactor* (r = 0.41), *Treponema* (r = 0.39), *Kingella* (r = -0.36), *Corynebacterium* (r = -0.34), *Porphyromonas* (r = 0.33), *Fretibacterium* (r = 0.32), and *Dialister* (r = 0.31). The direction of correlation for each bacterial genus was consistent across CAL, RBL (%), and BOP (%) in **Figure 6A**. The Correlation analysis between systemic inflammatory markers (WBC, NEUT, NLR, PLR, and SII) and the subgingival microbiota revealed that multiple periodontitis-associated bacterial genera exhibited significant positive correlations with these indicators (*P* < 0.05). Specifically, white blood cell count (WBC) was positively correlated with *Porphyromonas* (r = 0.26), *Treponema* (r = 0.31), *Fretibacterium* (r = 0.30), *Filifactor* (r = 0.28), and *Tannerella* (r = 0.19). NEUT showed a significant positive correlation with *Porphyromonas* (r = 0.32). Detailed correlation results for all systemic markers are presented in **Figure 6B**.

## 4 Discussion

Periodontitis is one of the most prevalent infectious diseases of the oral cavity, fundamentally characterized as a chronic inflammatory process driven by alterations in the composition and function of the subgingival microbiome. (Qian et al., 2021) As the primary etiological agent, the subgingival plaque microbiome maintains a dynamic equilibrium with the host under healthy conditions. However, once this balance is disrupted and dysbiosis ensues, it may trigger inflammatory responses in periodontal tissues. (Jang et al., 2025) With the rapid advancement of generation sequencing technologies, investigation into the pathogenesis of periodontitis has emerged as a focal point of research in oral microbiomics. Numerous studies have confirmed significant differences in the composition and structure of the oral microbiome between periodontitis patients and healthy individuals. (Kamer et al., 2024) Nevertheless, systematic and in-depth research on the regular patterns of succession of the subgingival microbiome during the progression of periodontitis, as well as its intrinsic relationship with disease development, remains lacking.

In this study, subgingival samples were collected from periodontally healthy controls and patients with periodontitis at various stages. Utilizing 16S rRNA sequencing technology, we systematically characterized the community structure and compositional features of the subgingival microbiome across different disease stages. These analyses provide direct evidence for understanding the structural and functional dysbiosis of the subgingival microbiome during periodontitis progression. Building upon this foundation, we further assessed microbial community diversity. Alpha diversity analysis was employed to evaluate species richness and evenness within each group, while beta diversity analysis was utilized to compare microbial community similarity or dissimilarity among groups. This comprehensive approach enabled characterization of the dynamic changes in the subgingival microbiome throughout periodontitis progression. The Comparison of alpha diversity indices between the healthy control group and periodontitis patients at different stages revealed that the Chao1, Pielou’s evenness, Shannon, and Faith’s phylogenetic diversity indices were all elevated in periodontitis patients across all stages relative to healthy controls. This finding suggests that subgingival microbial richness, diversity, and phylogenetic diversity are generally higher in periodontitis patients than in periodontally healthy individuals. Notably, microbial diversity did not increase linearly with disease severity but instead exhibited a dynamic trend of initial increase followed by decline. In early-stage periodontitis (Stages I–II), (Britos et al., 2025) microbial diversity increased markedly, which may be attributable to local environmental changes during the initial inflammatory response that promote the coexistence and proliferation of diverse bacterial species. However, as disease progressed to advanced stages (Stages III–IV), (Wang et al., 2025) microbial diversity decreased, reflecting profound alterations in the subgingival microenvironment. The formation of deep periodontal pockets substantially modifies ecological niches for multiple species, typically accompanied by specific pathogens (e.g., Porphyromonas and Treponema) gradually gaining dominance within the anaerobic environment of deep periodontal pockets. At this stage, the microbial community transitions from an early state of balanced symbiosis to a dysbiotic state driven by a limited number of pathogenic bacteria, whereby more adaptable pathogens supplant the original commensal flora. These dominant bacterial populations further exacerbate local inflammation and tissue destruction. This transition underscores the critical importance of early diagnosis and intervention in periodontal disease management. (He et al., 2025) Dynamic monitoring of changes in microbial diversity, in conjunction with clinical parameters such as probing pocket depth, provides valuable insights for assessing disease progression. (Xu et al., 2022) The findings of this study are consistent with previous reports, further confirming that subgingival microbial diversity is lower in periodontally healthy individuals than in periodontitis patients, and that diversity exhibits dynamic evolutionary patterns throughout the disease course. The Beta diversity analysis revealed significant alterations in subgingival microbial community structure with increasing periodontitis severity. By comparing microbial compositions across different disease stages, we could clearly observe the dynamic evolutionary trajectory of the microbial community during disease progression. (Jung et al., 2024) This not only enhances our understanding of subgingival microecological balance in healthy individuals but also provides potentially important insights for the diagnosis and treatment of periodontitis at different stages.

The study revealed that as periodontal disease progresses, microbial dysbiosis becomes progressively evident: the abundance of commensal bacteria such as *Neisseria, Haemophilus, Cardiobacterium*, and *Capnocytophaga* decreases, while that of opportunistic anaerobic bacteria including *Prevotella, Porphyromonas*, and *Treponema* increases. These ecological shifts resulting from alterations in microbial community abundance play a pivotal role in the progression of periodontal disease. The total relative abundance of the orange complex-comprising *Fusobacterium, Prevotella, Parvimonas*, and *Campylobacter*, which are associated with major periodontal pathogens-followed the pattern: control group < Stage III < Stage IV < Stage I = Stage II. The marked increase in the orange complex serves as a key microbiological indicator of the transition from early/mild periodontitis to irreversible severe periodontitis. (Dai et al., 2025) The red complex-comprising *Porphyromonas, Tannerella*, and *Treponema*, which are associated with the primary pathogens of periodontitis-was detected in all five groups, with total relative abundance following the pattern: control group < Stage I = Stage II < Stage III < Stage IV. Importantly, these two complexes do not operate independently but rather form a “destructive alliance.” The peptides and amino acids generated by the orange complex through degradation of tissue proteins provide essential nutrients for the growth of the red complex. (Pandian et al., 2023>) Additionally, the orange complex (particularly *Fusobacterium*) supplies adhesion sites for the red complex, and together they help maintain a hypoxic microenvironment. (Abu Fanas et al., 2021) Collectively, they activate distinct immune pathways, producing additive or synergistic destructive effects. (Qian et al., 2023) It is well established that pathogenic bacterial genera typically do not act as solitary pathogens; rather, the pathogenicity of the microbial community is determined by the interactions among multiple genera. Our findings further substantiate this theory. Significant intergroup differences were observed in the periodontitis-associated bacterial genera identified, including *Campylobacter, Porphyromonas, Treponema, Parvimonas, Tannerella*, and *Filifactor* are all exhibited significant intergroup differences. We further screened for genera that significantly contributed to the discrimination among the five groups and found that the control group was significantly enriched with *Capnocytophaga, Corynebacterium, Neisseria, Cardiobacterium, Streptococcus*, and *Actinomyces.* Previous studies have demonstrated that Cardiobacterium is significantly enriched in periodontally healthy individuals, and its reduction may increase the risk of periodontitis onset and progression. Under healthy conditions, the subgingival microbiota is dominated by aerobic and facultative anaerobic commensal bacteria, with opportunistic anaerobic bacteria present only at low abundances. In the Stage III and IV (severe periodontitis) groups, *Porphyromonas* and *Treponema* associated with the red complex were significantly enriched. As periodontitis progresses, periodontal pocket formation and the resulting local anaerobic environment intensify, leading to massive proliferation of these opportunistic anaerobes. In turn, it creates favorable conditions for colonization by the strictly anaerobic red complex, ultimately resulting in a pathogen-dominated ecological imbalance that drives local inflammation and tissue destruction (Zhu et al., 2025).

Correlation analysis revealed significant linear associations between the relative abundances of various subgingival bacterial genera and clinical periodontal parameters. Specifically, *Filifactor, Treponema, Porphyromonas, Fretibacterium, Dialister*, and *Tannerella* were positively correlated with periodontitis severity, whereas *Rothia, Cardiobacterium, Haemophilus, Kingella*, and *Corynebacterium* exhibited negative correlations. These findings further support the potential role of specific bacterial communities in the initiation and progression of periodontitis. (Bassani et al., 2023) Notably, *Treponema* and *Porphyromonas*, as core members of the “red complex,” were closely associated with clinical inflammatory indicators such as probing pocket depth and bleeding on probing. WBC and their subtypes are commonly employed as clinical markers of systemic inflammation and are widely utilized to assess the body’s immune and inflammatory responses. (Zare et al., 2023; Özdemir et al., 2024) During bacterial infections, different types of leukocytes interact to mediate the inflammatory process. Compared with individual blood cell parameters, composite inflammatory indices such as the NLR, PLR, and SII integrate information from multiple inflammation-related cell types. These composite markers are less susceptible to fluctuations caused by acute inflammation and offer greater stability, rendering them particularly suitable for evaluating chronic systemic inflammatory states. The present study demonstrated that, compared with patients with Stage I periodontitis, those with Stage IV periodontitis exhibited significantly elevated levels of WBC, NEUT, NLR, and SII, while no significant differences were observed in PLR or LMR. These findings are generally consistent with previous reports. Further correlation analysis between these inflammatory markers and the subgingival microbiota revealed that WBC, NEUT, NLR, and SII were significantly positively correlated with the relative abundances of *Porphyromonas, Treponema, Tannerella, Fretibacterium*, and *Filifactor*, suggesting that these periodontal pathogenic genera may have a potential association with the host’s systemic inflammatory response. (Sonets et al., 2025) These findings indicate that periodontitis-associated subgingival microbiota exhibit significant differences across disease stages, and these differences align with trends in systemic inflammatory marker levels, further supporting an association between local periodontal dysbiosis and systemic inflammation. Consequently, long-term uncontrolled periodontal infection and tissue destruction may subclinically increase the systemic inflammatory burden, thereby potentially elevating the risk of cardiovascular disease. (Mehrnia and Van Dyke, 2026) From a mechanistic perspective, this phenomenon may reflect a bidirectional interaction between local periodontal inflammation and microbial dysbiosis. (Adam et al., 2025) On one hand, periodontal inflammation creates favorable conditions for the overproliferation of anaerobic bacteria by providing tissue degradation products as a nutrient source and altering the subgingival redox environment. (Hascoët et al., 2026) On the other hand, microbial dysbiosis may further exacerbate local inflammation and periodontal tissue damage through activation of the host immune response, thereby establishing a self-perpetuating vicious cycle. (Lundmark et al., 2019) Therefore, these bacteria not only play a significant role in the onset and progression of periodontitis but may also contribute to the development and progression of systemic diseases by mediating systemic inflammatory pathways (Bhuyan et al., 2022).

This study employed 16S rRNA sequencing to systematically characterize the subgingival microbiome in periodontally healthy individuals and patients with periodontitis at different stages. In conjunction with the measurement of systemic inflammatory markers, we investigated the potential association between subgingival microbial community structure and systemic inflammatory status. Our findings provide novel insights into the dynamic changes in the subgingival microbiome during periodontitis progression and its correlation with systemic inflammation. Nevertheless, several limitations of this study should be acknowledged. First, the relatively modest sample size may have limited the statistical power of the 16S rRNA sequencing and inflammatory marker analyses; future studies with larger cohorts are warranted to validate the current findings and thereby improve the reliability of the results. Second, as a single-center case-control study, this research identified an association between the subgingival microbiome and systemic inflammation but cannot establish causality. Furthermore, although we controlled for potential confounding factors by matching participants for age, sex, and BMI, other variables such as dietary habits and geographical location were not accounted for, despite their potential influence on microbial community composition and systemic inflammatory regulation. It should be noted that the systemic inflammation markers employed in this study are routine hematological parameters, which are generally considered nonspecific indicators of inflammation. Although these markers are commonly tested and clinically significant, from the perspective of clinical translation, they offer advantages, including low cost and direct accessibility from blood test reports. Nevertheless, future studies should incorporate more comprehensive and specific inflammatory markers, such as the cytokines interleukin−6 (IL−6), interleukin−1β (IL−1β), tumor necrosis factor−α (TNF−α), and interleukin−10 (IL−10). Incorporating such markers would enable a more precise assessment of the relationship between the subgingival microbiome and systemic inflammation. Finally, it is worth noting that hormones have been shown to influence the oral microbiome and inflammatory responses. Nevertheless, given that hormone levels were not measured in this study, their potential impact on the findings cannot be ruled out.

Future research should involve large scale, multicenter prospective cohort studies that incorporate a broader range of specific inflammatory markers to validate these findings and explore causal relationships. Concurrently, integrating *in vivo* and *in vitro* functional experiments will help elucidate the molecular mechanisms through which periodontitis exacerbates systemic chronic inflammation. Finally, the application of multi-omics approaches, such as metagenomics and metabolomics, will further elucidate the complex interplay between the subgingival microbiota and host systemic inflammation, potentially identifying microbial targets for risk assessment and precision intervention in systemic diseases. Furthermore, given the limitation that hormone levels were not assessed in the present study, future research should conduct longitudinal studies exclusively in female populations to examine hormone levels across menstrual cycle phases, as well as the association between hormonal medication use and subgingival microbiome structure. Such studies would enhance our understanding of hormones’ role in periodontitis-associated systemic inflammation and inform the development of personalized treatment strategies.

## 5 Conclusion

Through systematic analysis of subgingival microbiota composition and systemic inflammatory marker levels in patients with periodontitis at different stages, this study confirmed the presence of dynamic dysbiosis in the structure and function of the subgingival microbiome in periodontitis patients, accompanied by significantly elevated systemic inflammatory levels. Furthermore, the findings revealed a positive correlation between the phased succession patterns of specific subgingival microbial communities during periodontitis progression and systemic inflammatory markers. This provides cross-disciplinary evidence from microbiological and immunological perspectives to elucidate the potential “inflammatory link” mechanism connecting periodontal infection with systemic diseases such as cardiovascular disease. Collectively, these findings suggest that regulating the subgingival microbiome or dynamically monitoring specific microbial succession patterns may represent a potential strategy for assessing the risk of periodontitis-related systemic inflammation, enabling early warning, and facilitating precision intervention.

## Data Availability

The data presented in this study are deposited in the National Center for Biotechnology Information (NCBI) Sequence Read Archive (SRA) repository, accession number PRJNA1470369.
